# A remote Digital Twin interaction system based on virtual mobile infrastructure

**DOI:** 10.1038/s41598-026-50381-w

**Published:** 2026-04-29

**Authors:** Zhipeng Fu, Jun Zhou, Wanpeng Xu

**Affiliations:** 1 School of Computer Science and Technology, Guangdong University of Technology, Guangzhou, 510006 Guangdong China; 2 Industrial Internet Institute, New-Typeype Network Research Department, Peng Cheng Laboratory, Shenzhen, 518055 Guangdong China; 3 The School of Computer Science and Engineering, Sun Yat-Sen University, Guangzhou, 510006 China

**Keywords:** Digital Twin, GPU, Remote, Virtual mobile infrastructure, Mobile terminal, Engineering, Mathematics and computing

## Abstract

To address the challenge of achieving smooth real-time presentation and interaction of factory Digital Twin (DT) systems on mobile terminals, this paper proposes a Virtual Mobile Infrastructure (VMI) based Digital Twin Remote Interaction System (VDTRIS). It improves the performance of DT applications in real-time presentation and interaction on mobile devices by making the following two key improvements to the conventional VMI. First, the transmission path has been optimized by eliminating unnecessary links (e.g. OpenGL ES transmission). Concurrently, Android Docker has the capacity to access GPU resources directly by developing a mobile GPU driver. Second, integrating a dedicated Video Processing Unit (VPU) to specifically handle the RGBA coding of GPU-rendered results into H.264 format directly, thereby reducing data transmission in RGBA format and offloading CPU coding tasks that consume substantial CPU resources. These improvements collectively reduce transmission latency and CPU consumption, leading to more efficient real-time presentation and interaction on mobile terminals. Experimental results show that VDTRIS performs better than existing state-of-the-art schemes, particularly on mobile terminals with insufficient hardware configurations. By adopting VDTRIS, factory DT applications can effectively overcome constraints like geographical restrictions, insufficient computing resources of mobile terminals, and limited funds. Meanwhile, VDTRIS can be easily extended to other digital twin application fields and resource-intensive applications.

## Introduction

With the development of intelligent manufacturing, Internet of Things (IoT) and Industrial Internet of Things (IIoT), more and more manufacturing enterprises hope to transmit the real-time status of production line and production equipment on the office computers, tablets, and mobile phones through the network, so that factory leaders, production line managers, and maintenance man can watch and monitor the working status of production lines and equipment anytime and anywhere^1–3^. Digital Twin (DT) is a virtual model that uses digital technology to carry out real-time mapping and simulation of physical objects, systems, or processes in the digital space. It can use technical means such as sensors, IoT, big data, and artificial intelligence (AI) to collect data on the three-dimensional appearance and operation status of physical objects in real time, and carry out analysis, prediction, and optimization in a virtual environment, so as to achieve comprehensive perception, understanding, and control of physical objects.

The application of DT technology for round-the-clock remote real-time monitoring, analysis, and operation maintenance of factories and their equipment is becoming increasingly popular^1,4^. DT technology is involved in various aspects, from the manufacturing^5^, design^6^, operation maintenance^7^, fault diagnosis^8^, remaining useful life prediction^9^, and assembly error analysis of equipment^10^, to the visual monitoring of production workshops^2^ and the application of DT in production lines^11^. However, since DT requires substantial general computing, graph computing, storage, and other resources, DT systems are often implemented in the cloud. At the terminal, a PC with good performance is also required to achieve real-time presentation and smooth communication of 3D images.

With the iterative development of communication technologies, the Internet of Things, and pervasive computing, although mobile terminals such as smart phone and tablet are not as powerful as PCs in terms of storage and computing, their advantages of portability, real-time communication, popularity, and personalization have made people increasingly hope to use mobile terminals for round-the-clock real-time monitoring and operation maintenance of factories and equipment with DT technology. However, real-time presentation and interaction of equipment based on DT in mobile environments face the following problems:

- The computing and storage resources of mobile devices are limited, while DT systems require substantial computing and storage resources for real-time 3D presentation, real-time rendering, and other operations, which often cannot run smoothly on mobile devices.
- Since one of the main functions of DT is to perform real-time 3D presentation of production lines or equipment, this requires significant GPU computing power, which is often unavailable on ordinary mobile devices such as smartphones and tablets.

In summary, due to the limited computing and storage performance of mobile devices, there are significant challenges in the real-time presentation and interaction of DT systems on mobile terminals.

To solve the issue of limited performance of DT systems on mobile terminals, researchers have proposed several solutions. The common feature of these methods is that they offload and optimize computing and storage requirements at the edge to reduce the pressure on themselves. Although they partially address the problem of limited computing and storage performance at the edge, they do not propose effective solutions for real-time 3D image rendering and presentation in DT systems on mobile and edge terminals. This paper proposes a VMI-based Digital Twin Remote Interaction System (VDTRIS) in a computing power network.

With the development and popularization of cloud computing, big data, and artificial intelligence, the types and capabilities of computing demands have become increasingly diversified. Not only has general-purpose computing emerged, but various forms, such as Graphic Processing Unit (GPU) computing, Tensor Processing Unit (TPU) computing, Deep learning Processing Unit (DPU) computing and Neural network Processing Unit (NPU) computing, have also emerged. Meanwhile, the demand for computing power includes not only large-scale supercomputing for meteorology, aerospace, petroleum, etc., but also small-scale computing and micro-computing for enterprises and individuals.

To meet the increasingly diverse computing needs and enable people to use computing power resources like water and electricity, scientists have proposed the concept of a computing power network^12–15^. It connects distributed computing nodes, realizes the management and allocation of computing power resources such as computing, storage, and networks through the network control plane, and presents them to thousands of households via the Internet. The computing power network intelligently schedules heterogeneous computing resources distributed at terminals, edges, and clouds by real-time sensing of user task requirements, network conditions, and resource status of computing nodes.

Virtual Mobile Infrastructure (VMI) is one of the architectures of the computing power network. It refers to virtualize a mobile operating system on cloud servers, and on mobile terminals, users can remotely connect to and interact with the system through VMI client software^16–18^. Diverse resources of the computing power network, such as CPU, GPU, NPU, and storage, can all become part of the resources of the VMI, which provides beneficial support for mobile users to access the computing power network via VMI.

However, existing VMI solutions face three challenges that impede system performance. First, high transmission latency significantly degrades real-time 3D presenting performance. Second, the absence of commercial-grade GPU drivers optimized for mobile environments prohibits direct invocation of cloud-based GPU resources. Third, open-source driver implementations lack sufficient coding capability to handle 3D image rendering and data coding.

To mitigate transmission latency and minimize CPU resource consumption, VDTRIS incorporates two key improvements over conventional VMI systems: a custom Graphics Processing Unit (GPU) driver is developed to enable direct GPU resource invocation for rendering, eliminating multi-stage instruction transmission from DT server to remote mobile terminals. Second, a dedicated Video Processing Unit (VPU) is integrated into the system to encode the output image in H.264 format, replacing CPU encoding which consumes massive CPU resources.

The main idea of VDTRIS is as follows: On the high-performance servers at the central nodes of the computing power network, we simultaneously run the DT system connected to the factory and the server application of VMI, which starts Android Docker. The terminal presentation software of the DT system is installed on Android Docker. The content is then transmitted through wired/wireless networks and displayed on multiple mobile intelligent terminals. For 3D image Rendering, VDTRIS invokes the mobile GPU driver directly in Android Docker. All 3D image processing is performed on the virtual mobile operating system at the central nodes of the computing power network, fully leveraging the advantages of these nodes in high computing performance and powerful functionality. Mobile terminals are only used for display and interaction, avoiding the performance bottleneck caused by limited terminal capabilities that hinder timely and efficient processing of real-time, large-scale graph calculations in the DT system. This makes it possible to achieve real-time presentation and interaction of DTs on mobile intelligent terminals.

The main contributions of this paper are as follows:

- Propose a VMI-based digital twin remote interaction system suitable for mobile terminals to use cloud resources for real-time presentation and interaction with cloud DT systems.
- Improve the method for efficiently using server GPUs in virtual Android systems.
- To offload the CPU workload and improve system rendering efficiency, propose a method of adding a VPU to replace the CPU for 3D image rendering and encoding.

The remaining content of this paper is organized as follows: Sect. "Literature review" presents a systematic review of existing relevant literature; Sect. "VMI-based Digital Twin remote interaction system (VDTRIS)" elaborates on the system architecture and working process of VDTRIS in detail; Sect. "Improvements in the VDTRIS" focuses on explaining the improvements made by VDTRIS; Sect. "Performance experiment" verifies the system through performance comparison experiments; Sect. "Discussion and future work" conducts an in-depth discussion on the system’s characteristics, limitations, and future development prospects; finally, Sect. "Conclusion" provides a summary of the research.

## Literature review

### Computing power network (CPN)

The concept of Computing Power Network (CPN) has emerged to integrate distributed computing resources (cloud, edge, terminal) and enable efficient resource orchestration^12–15^. CPN provides a foundational infrastructure for applications requiring high computation, such as Digital Twins (DT), by dynamically allocating resources like GPU and storage.

Typical CPN architectures adopt a layered structure integrating cloud, edge, and terminal resources^13–15,19,22^. For instance, Lei Bo et al.^22^ proposed a computing power network architecture integrating cloud computing nodes, edge computing nodes, and terminal nodes, with a management and orchestration system for computing, storage, and network resources. Such architectures support flexible resource sharing and task offloading, which is relevant to the remote rendering approach proposed in this paper.

VMI is one architectural realization of CPN, enabling mobile devices to leverage remote cloud resources by hosting a virtualized mobile OS on servers^16–18^. Our work builds upon VMI to specifically address GPU-intensive rendering for DT applications.

### Presentation or interaction for mobile Digital Twins

To address the resource limitations of mobile devices in running DT systems, scholars have proposed solutions primarily based on edge computing task offloading. These approaches offload computing, storage, and rendering tasks from mobile devices to infrastructure such as base stations and servers close to edge nodes. For example, Bozkaya^26^ proposed a DT framework for edge service location selection in mobile edge computing environments. Zheng et al.^27^ proposed a novel Digital-Twin Cloud-Edge Networks (DTCEN) model, which offloads and optimizes the computing and storage services at the edge, thereby improving resource utilization and optimizing the load of edge devices. Kuang et al.^28^ and Zhou et al.^29^ proposed task offloading methods based on deep reinforcement learning. Song et al.^30^ proposed a cache-based computing offloading method for multi-region mobile edge networks.

The common feature of these methods is that they offload and optimize the computing and storage requirements at the edge, thereby reducing the computational and storage pressure on edge devices. While effective for general computation, these approaches often do not provide specialized optimization for real-time 3D graphics rendering and low-latency interaction, which are critical for DT visualization.

### VMI solutions

VMI technology offers a promising approach to address computing limitations in mobile devices, with recent studies focusing on performance optimization. Liu et al.^31^ developed cMobiDesk, a lightweight VMI platform using Linux Containers to create Android instances without modifying the OS kernel. Wang et al.^32^ introduced FUSION, a Unified Application Model, with distinguishing IPC (Inter Process Communication) events between local and non-local resource access, to solve the communication issues between identical applications on the remote VMI server and the local device caused by the separate installation of the same application. Choi et al.^33^ improved KVM hyper call mechanisms to reduce host CPU load during large-scale service operations. Fu et al.^34,35^ presented a VMI solution on Telemedicine. Su et al.^36^ optimized vMobiDesk for virtualized data transmission, enabling low-overhead remote input, audio, and camera redirection.

Current VMI solutions primarily optimize transmission efficiency and resource management^32,33,36^. However, there is limited research on enabling efficient 3D image rendering with VMI in mobile environments due to two key challenges. First, the lack of commercial GPU drivers for mobile devices forces most rendering tasks to rely on servers/PCs. Second, the insufficient capabilities of open-source drivers to concurrently handle 3D rendering and video encoding. Our work directly addresses these gaps.

### Cloud graphics streaming

Our approach is also related to cloud graphics streaming technologies, such as Cloud Gaming and Pixel Streaming^37,38^, which render graphics on remote servers and stream the video to thin clients.

Cloud Gaming platforms like NVIDIA CloudXR^39^ and Google Stadia leverage powerful cloud GPUs to render games and stream compressed video frames to user devices, focusing on minimizing end-to-end latency^37^. Similarly, Pixel Streaming technology (e.g., in Unreal Engine^40^) encodes GPU-rendered frames in real-time and transmits them via WebRTC, enabling high-fidelity 3D applications to run in browsers.

While these technologies share the core principle of remote rendering with VDTRIS, they are generally designed for desktop or gaming scenarios and are not optimized for the Docker-containerized Android environment and industrial DT applications with specific interaction needs. Furthermore, they often rely on standard GPU passthrough or API forwarding, unlike our integrated approach of a custom GPU driver and dedicated VPU encoding within a VMI framework. A comparison of key characteristics is summarized in Table 1.

**Table 1:** comparison of remote graphics rendering approaches.

Feature	Cloud gaming	Pixel STREAMING	VDTRIS
Target application	Games, entertainment	3D visualization, simulation	Industrial Digital Twins
Client environment	Native application, browser	Browser (WebRTC)	Android/iOS/H5 App (VMI Client)
Rendering environment	Windows/Linux VM, direct GPU access	Windows server, GPU	Android docker, custom GPU driver
Encoding	GPU (NVENC)/CPU	GPU (NVENC)/ Software	Dedicated VPU
Primary optimization	Low latency for gaming	High visual fidelity	Mobile DT interaction efficiency

## VMI-based Digital Twin remote interaction system (VDTRIS)

### The system topological structure

**Fig. 1:**
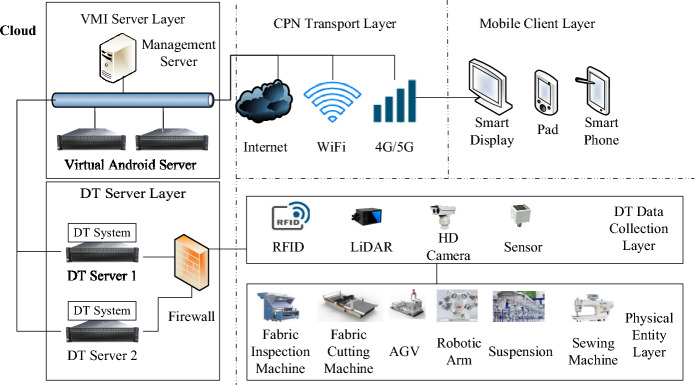
The topological structure of the VDTRIS system.

The VDTRIS topological structure consists of six layers, as shown in Fig. 1. There are the Physical Entity Layer, DT Data Collection Layer, DT Server Layer, VMI Server Layer, Computing Power Network (CPN) Transport Layer, and Mobile Client Layer. Each layer is connected via wired or wireless networks.

The Physical Entity Layer primarily consists of the physical production line equipment in the factory. For the garment production line, it mainly includes fabric inspecting machines, fabric cutting machines, AGV vehicles, robotic arms, suspension systems, sewing machines, etc. These devices are connected to form a garment production line through automated or semi-automated means.

The DT Data Collection Layer is mainly used to collect three-dimensional parameters of various devices in the Physical Entity Layer, such as their appearance, working status, operational actions, and movement directions. Additionally, this layer can also collect other parameters like temperature and rotational speed. It primarily includes RFID, LiDAR, high-definition cameras, sensors, etc. These devices transmit the collected information to the DT system, providing data support for the system’s real-time presentation and remote virtual-real interaction.

The DT Server Layer: This layer mainly includes servers equipped with the DT system and firewalls. Since the DT system is primarily a server-side system, it requires high-performance servers with advanced functionalities.

The VMI Server Layer: This layer mainly consists of VMI servers, including VMI management servers, VMI storage servers, and firewalls. The VMI system is also stored on this layer, which is described in Section "The typical structure of the VMI system on VMI server layer". The VMI management server is responsible for the unified management of VMI storage servers. According to task requirements, it virtualizes the server into multiple Android Docker containers on the VMI storage servers. Each Android Docker acts as a virtual mobile phone, running the mobile DT application. This allows the virtual Android system to leverage the powerful computing and storage capacity of the server side, enabling the smooth operation of the mobile DT application and interactive capabilities.

The CPN Transport Layer: This layer establishes network transmission between the VMI server side and mobile clients, mainly including network transmission methods such as Internet to WIFI and 5G.

The Mobile Client Layer: This layer serves as the receiving layer for VMI virtual clients, including smart displays, tablets, smartphones, and other intelligent terminals.

### The typical structure of the VMI system on VMI server layer

The VMI Server Layer is primarily used for building and implementing a VMI-based virtual Android application framework. Existing VMI schemes mainly use Xen^36^, VirtualBox^36^, Linux Container^31^, or KVM^32,33,36^, few use Docker. A major limitation of these approaches is that, due to their dependence on virtual machine–based architectures, the server side tends to be heavily loaded. Both system startup and runtime operations consume substantial CPU resources, and the initialization time is relatively long. To enable the Android operating system to run in Docker containers, which significantly reduces server-side overhead and enables faster startup, this paper introduces Ashmem and Multi-Instance Binder into the cloud operating system. Ashmem, which means Anonymous Shared Memory, is used by the Android system to implement memory sharing. Multi-Instance Binder is a service process that enables communication between different Android processes in the cloud operating system.

**Fig. 2:**
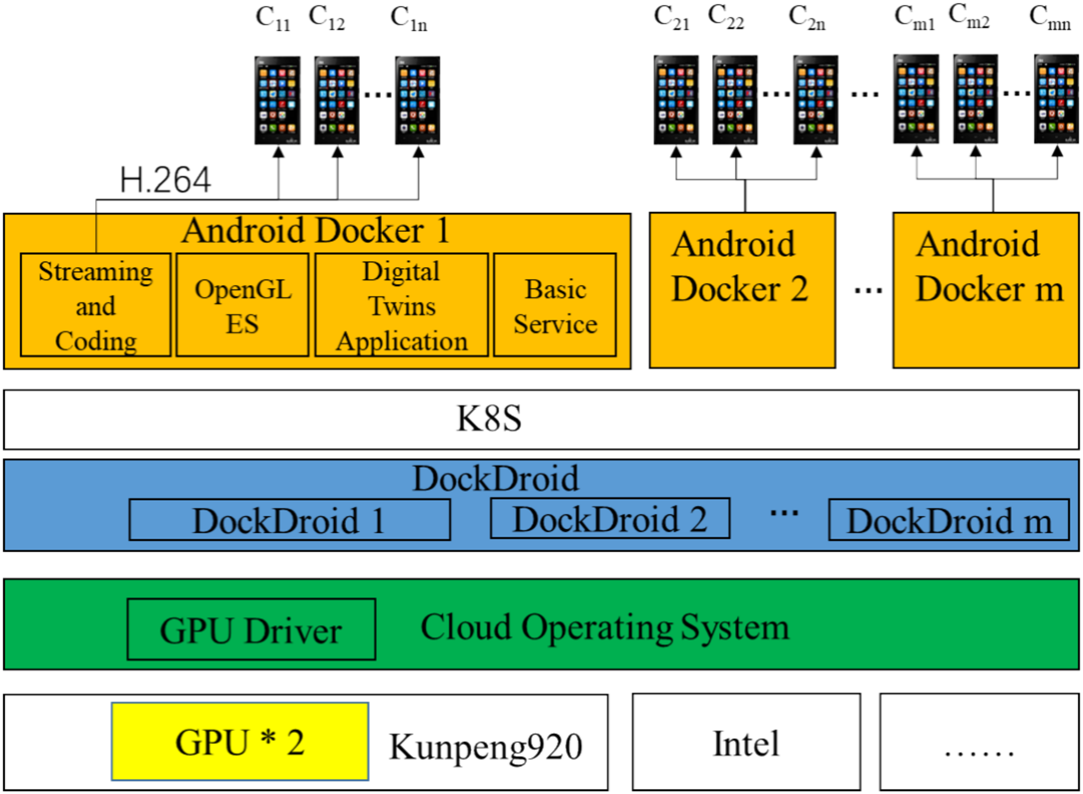
The framework structure of the vmi system on VMI server.

As illustrated in Fig. 2, the framework structure of the VMI system on the VMI Server Layer is divided into six layers. From bottom to top consists of the physical server layer, the Cloud Operating System layer, the DockDroid layer, the K8S layer, the Android Docker layer and the client SDK layer. The detailed functions of these Layers are descripted as follows:

The Physical Server Layer is a cluster composed of various servers, which is part of the computing power network. It can include servers from Intel, Phytium, Huawei Kunpeng, Ascend, etc. These servers together form a VMI server cluster and work as part of the computing power network, providing services such as general computing, GPU computing, storage, and so on.

The Cloud Operating System Layer centrally manages the underlying physical servers, masks the heterogeneity of the underlying physical servers, and provides a unified basic software environment upwards. Modules such as Multi-instance Binder and Ashmem are added to the system kernel to support the operation of the Virtual Android environment. Traditionally, GPU drivers are installed at this layer.

The DockDroid Layer consists of multiple DockDroid processes. Each DockDroid process corresponds to an Android Docker. The main functions of DockDroid are as follows: first, it is responsible for getting OpenGL ES instructions from Android Docker; second, it translates these instructions from OpenGL ES into OpenGL that can be recognized and executed by GPU drivers; third, it handles data transmission from the cloud operating system back to Android Docker.

The K8S Layer is implemented based on an open-source solution, compatible with standard K8S interfaces. It is used to create, manage and allocate the Android Docker.

The Android Docker Layer is composed of multiple Android Docker, which provides an Android runtime environment, enabling Android applications to run smoothly within the Android Docker. Each Android Docker includes the following four modules: the Streaming and Coding Module, OpenGL ES Module, Digital Twins application module and Basic Service Module, as shown in Fig. 2. These modules enable the Android Docker to provide capabilities such as image rendering, stream processing, image encoding/decoding, and display services.

- The Streaming and Coding Module is used to receive the rendered images, code them from RGBA to H.264, and transmit the coded results to the client.
- The OpenGL ES Module primarily handles the instruction recognition for mobile devices. The OpenGL ES API is a subset of the OpenGL standard specification, specifically tailored for mobile/embedded environments. It provides a standard specification for researchers and engineers to implement rendering functions in the Android system. This paper implements the subset standard and places it in the dynamic library libGL_**.so DLL.
- Digital Twin Application Module: The Digital Twin Application Module serves as the client of the DT system. It enables the DT system to be presented in real time on mobile terminals and facilitates real-time interaction between mobile terminals and the system.
- The Basic Service Module provides the basic runtime environment services for Docker.

The Client SDK Layer. Currently, mobile devices are mainly divided into three categories: Android, Apple’s iOS systems, and HTML5 (H5) systems for smart screens. Therefore, this paper provides corresponding SDKs for these three types of clients, mainly including Android SDK, iOS SDK, and H5 SDK. When users access the VMI system using an iPhone, the corresponding iOS SDK version will be installed on the user’s iPhone.

### The typical entire workflow of the VDTRIS

**Fig. 3:**
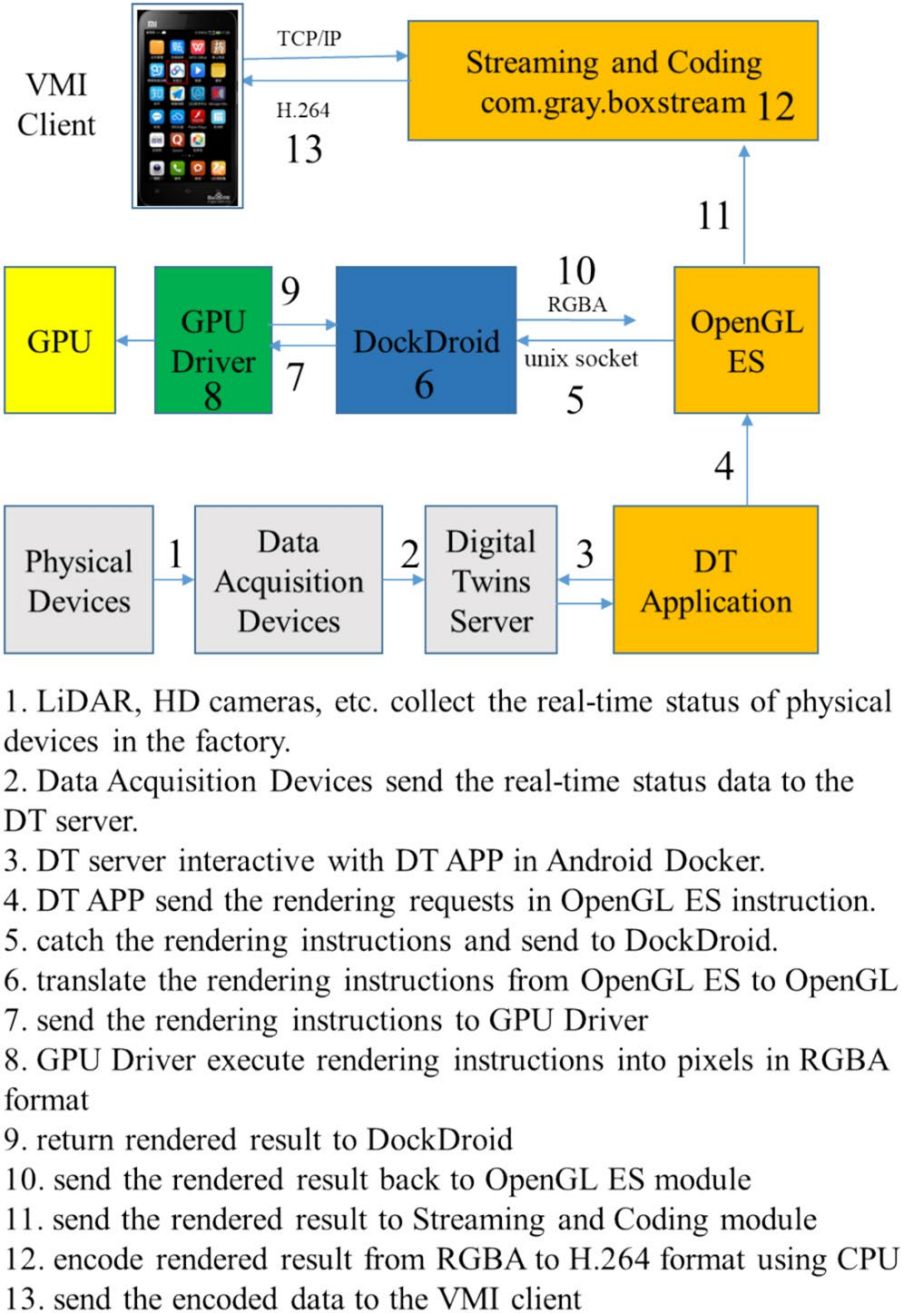
The entire workflow of the VDTRIS.

We can divide the entire workflow of the VDTRIS into two segments: DT System interaction between physical equipment and the DT application, and the DT application presented to the remote mobile client. Workflow processes 1–3 in Fig. 3 shows the first segment. The garment production line equipment in the Physical Entity Layer operates normally. Its status and 3D device information are acquired by the DT Data Collection Layer through LiDAR, high-definition cameras, etc., and then subjected to the Digital Twin system in the Digital Twin Server Layer. The DT application in the Android Docker interacts in real-time with the DT system, both 3D presentation and real-time rendering of the DT application are completed by the VMI server.

The second segment of the VDTRIS is to present the DT application image (or 3D image) in the Android Docker. Its workflow is processes 4–13 as shown in Fig. 3. The DT application sends the rendering requests in OpenGL ES instruction to the OpenGL ES module. Then the instructions is transmitted from the OpenGL ES module to DockDroid via a Unix socket. After that, it will be translated into OpenGL instructions and then sent to the GPU driver to execute.

After the GPU completes rendering, the results are presented as pixel data in RGBA format. This data will be transmitted via DockDroid and OpenGL ES module to the Streaming and Coding module, where it is encoded from RGBA to H.264 format. Finally, it is sent to the VMI client terminal.

## Improvements in the VDTRIS

### OpenGL ES transmission omitted and direct GPU invocation

Figure 3 shows that the workflow of the VDTRIS involves 13 steps crossing multiple modules in different layers, which cause a high transmission delay. And too many transmission steps might also overburden the CPU.

From the workflow, we can see that the rendered results are transmitted from DockDroid to the Streaming and Coding module via OpenGL ES. To reduce the transmission latency and CPU overhead caused by multiple data copies and context switches, we omit the OpenGL ES transmission step. Instead, we modify the DockDroid to directly pass the rendered result to the Streaming and Coding module. This is achieved by Unix Domain Socket technology between the DockDroid process and the Streaming and Coding module in Android Docker.

To further the research, we tested the frame rate (calculated in FPS, frames per second) of rendering results displayed on client devices and the CPU utilization rate of DockDroid for two scenarios: an unimproved scheme of transmission with the OpenGL ES module and an improved scheme of transmission excluding the OpenGL ES module. The FPS upper limit was set to 60 FPS. Performance parameters were tracked via Perfdog, an FPS performance testing and analysis tool. Theoretically, the ideal scheme features a higher FPS value and a lower CPU utilization rate.

The testing equipment was a Huawei Kunpeng dual-CPU server, including 48*2 CPU cores, 512 GB RAM, 1 TB SSD, 8 TB SATA hard disk, and AMD Radeon W6800 GPU. The 96 cores were allocated sequentially from 0 to 95. We repeat the comparison experiments eight times under conditions where different numbers of CPU cores were allocated to DockDroid and Android Docker.

The comparison test results associated with each core number setting are shown in Table 2. The FPS of the unimproved scheme was consistently lower at about 8%−23% compared to the improved scheme. Because the amount of tasks to be processed within the DockDroid is not significantly different between the two schemes, but CPU utilization in DockDroid was about 8%−34% lower in the unimproved VMI scheme, suggesting that its data transmission process consumes more CPU resources, such that less CPU resources are available for DockDroid.

**Table 2:** Table of two data transmission schemes comparison.

Test No	CPU core number in android docker	CPU core number in DockDroid	Scheme type	Steam fetching FPS	CPU utilization of DockDroid
1	2^3,4^	1^19^	Unimproved	43	53%
			Improved	55	66%
2	2^3,4^	2^19,20^	Unimproved	45	56%
			Improved	58	79%
3	3^4–6^	1^4^	Unimproved	41	46%
			Improved	51	70%
4	3^4–6^	1^19^	Unimproved	46	53%
			Improved	59	69%
5	3^4–6^	2^20,21^	Unimproved	51	63%
			Improved	56	77%
6	4^3–6^	1^21^	Unimproved	50	69%
			Improved	58	73%
7	4^3–6^	2^20,21^	Unimproved	51	78%
			Improved	58	81%
8	4^3–6^	1^5^	Unimproved	51	66%
			Improved	59	74%

Test results above suggest that stream fetching excluding the OpenGL ES module is more desirable in FPS and overall CPU performance. As such, instead of rendered data transmission from DockDroid through OpenGL ES to the Streaming and Coding module as designed in a typical VMI-based solution, the improved VDTRIS solution proposed by this paper adopts an improved scheme which will have the Streaming and Coding module fetch stream data directly from DockDroid.

In the conventional design of VMI, the function of DockDroid is to receive the render request from OpenGL ES module, translate it from OpenGL ES into OpenGL, and then send it to the GPU driver to execute, as shown in Fig. 3. After that, DockDroid gets the rendered results from the GPU driver and sends them to the Streaming Service module. This scheme brings more communication overhead and significant CPU consumption to translate. Because when displaying the same rendered image frame, the RGBA format data is much larger than H.264 format data.

Since the Android system does not have official GPU drivers, Android Docker needs to leverage the cloud operating system to access GPU resources. For this reason, this paper develops a mobile GPU driver for Android Docker so that GPU in the hardware layer can be invoked directly. This mobile GPU driver is a complete native driver implementation, rather than an API forwarding or wrapper mechanism implemented inside the container. The driver is implemented as a user-space driver that communicates with the host GPU via the VFIO (Virtual Function I/O) GPU passthrough interfaces. The detailed implementation is as follows:

1. Driver Architecture: Our mobile GPU driver consists of two parts: a frontend driver in the Android Docker and a backend driver in the host. The frontend driver presents a standard OpenGL ES interface to the Android applications. Its main function is to make the GPU recognize the OpenGL ES instruction, so that the instruction can be executed directly in Android Docker. When an application calls an OpenGL ES function, the frontend driver converts the OpenGL ES instruction into an OpenGL command and sends it to the backend driver via a virtqueue. The backend driver then executes the command on the physical GPU.
2. GPU Passthrough Configuration: We use the VFIO framework to passthrough a dedicated GPU to the Android Docker. This gives the Android Docker direct access to the GPU hardware, bypassing the host GPU driver. We also support the VirtIO-GPU method for sharing a GPU among multiple Android Dockers.
3. Memory Management: We use the DMA-BUF sharing mechanism to share the rendered frame buffer between the GPU and the Streaming and Coding module. This avoids copying the frame buffer back to the Android Docker’s user space.

By invoking the GPU directly from the Android Docker, we eliminate the DockDroid translation step and the associated CPU overhead. Hence, the No. 6, 7 and 10 steps of the workflow in Fig. 3, used for instruction data transmission and translation through DockDroid, are no longer needed. Then the DockDroid module can be eliminated.

**Fig. 4:**
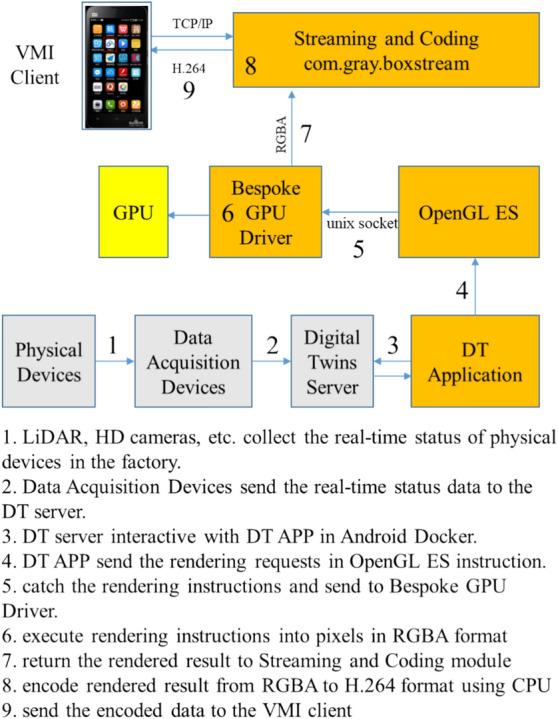
The new workflow with dockdroid omitted.

The improved workflow of the VDTRIS is demonstrated in Fig. 4. The DT application in the Android Docker get the real time DT 3D images through workflow processes 1–3, and the DT application represents 3D images to the remote VMI client through workflow processes 4–9.

### VPU coding

**Fig. 5:**
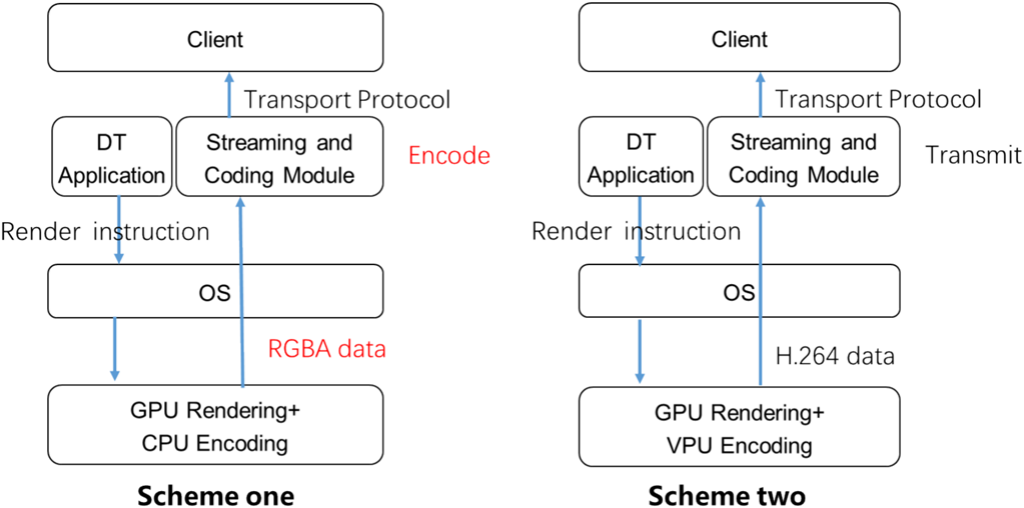
A comparison of workflows between CPU encoding and VPU encoding.

In traditional VMI solution, the DT frame needs to be rendered and coded so that it is suitable for transmitting and displaying on mobile devices before it is sent to the mobile terminal. However, whether the open-source GPU drivers or the mobile GPU drivers we’ve developed, neither is powerful enough to handle encoding the rendering results from RGBA format to H.264 format. Such that an alternative coding method is often required. A combination of GPU rendering and CPU coding is often adopted, as shown in Scheme 1, Fig. 5. In this compromised solution, rendered results will be sent from the GPU Driver to the Streaming and Coding module in Android Docker in RGBA format. And then they will be subsequently encoded into H.264 data by invoking CPU to execute. This solution has three disadvantages.

- RGBA coding generates larger size data compared to H.264 coding.
- RGBA coding add to more CPU load. CPU load will further increase when the RGBA data is converted into H.264 data by the Streaming and Coding Module.
- The solution is associated with a heavier transmit load hence greater transmission delay.

In order to improve the data coding performance, the VDTRIS system adds a VPU for frame coding instead of CPU coding, as detailed in Scheme 2, Fig. 5. Additionally, the rendered results are directly coded in H.264 format before transmitting to Stream and Coding module, shrinking data size and reducing transmission delay. Also, the VPU coding method improves the system performance by freeing up CPU overload.

The detailed implementation of VPU coding involves the following steps:

1. VPU Hardware Integration: We use a hardware VPU (e.g., Netint T432) that is installed in the VMI server. The VPU is exposed to the Android Docker as a PCIe device.
2. VPU Passthrough Configuration: We use VFIO to passthrough the VPU to the Android Docker, allowing the Android Docker to directly control the VPU.
3. API Developed: A user-space library is developed that implements the standard video encoding interfaces. Using this library the OpenGL ES module will send the encoding and rendering instructions to GPU and VPU hardware, and the rendered results will be encoded to H.264 directly.
4. Shared Memory Management: The shared frame buffer between GPU and the Streaming and Coding module in Section "VPU coding" is also shared with the VPU. The VPU directly reads the frame buffer from the GPU memory and encodes it into H.264. This avoids copying the frame buffer to the CPU memory.

To evaluate the performance of these two coding methods, we conducted a comparative experiment across servers with different specifications, measuring CPU and GPU utilization under each coding scheme. The servers involved were a Phytium model with a single 64-core CPU and a Huawei Kunpeng server featuring dual 48-core CPUs. The Netint T432 VPU was selected as the VPU coding hardware. CPU utilization was calculated based on a single core. When CPU demands surpassed a single core’s processing capacity, the CPU utilization would exceed 100%. Each experimental group was repeated 8 times, with the average results presented in Table 3. The results show that when using CPU coding, the CPU utilization of Phytium server and Kunpeng server reached 163% and 106% respectively. In contrast, with VPU coding, CPU utilization dropped to just 22.0 ± 1.8% and 9.1 ± 0.9% respectively—representing only 13.5% and 8.5% of the CPU-based scheme. This demonstrates that VPU coding significantly reduces CPU load.

**Table 3:** CPU coding VS VPU coding.

Server	64-core phytium server		48-core* 2 kunpeng server	
GPU	Tesla T4		AMD WX5100	
Coding	CPU coding	VPU coding	CPU coding	VPU coding
CPU utilization	163.3 ± 11.4%	22.0 ± 1.8%	106.4 ± 8.5%	9.1 ± 0.9%
GPU utilization	33.1 ± 2.6%	19.3 ± 1.5%	3.2 ± 0.3%	3.0 ± 0.3%

Additionally, the comparison of GPU utilization in Table 3 also indicates that VPU coding also substantially drop down GPU utilization. This is because in the CPU coding scenario, the GPU must wait for the CPU to finish copying and processing the previous frame before it can proceed with encoding the next one, leading to a congested pipeline and higher GPU utilization due to idle/wait states within its workload. The dedicated VPU, operating in parallel with the GPU, asynchronously handles encoding the moment a frame is ready. This allows the GPU to complete its rendering tasks more efficiently and proceed to the next frame without being stalled by the encoding process. Consequently, the GPU completes its core task faster and enters a lower-power state sooner, reflecting as lower utilization in our measurements.

Overall, adding VPU for rendered result encoding markedly improves the performance of the VDTRIS system.

**Fig. 6:**
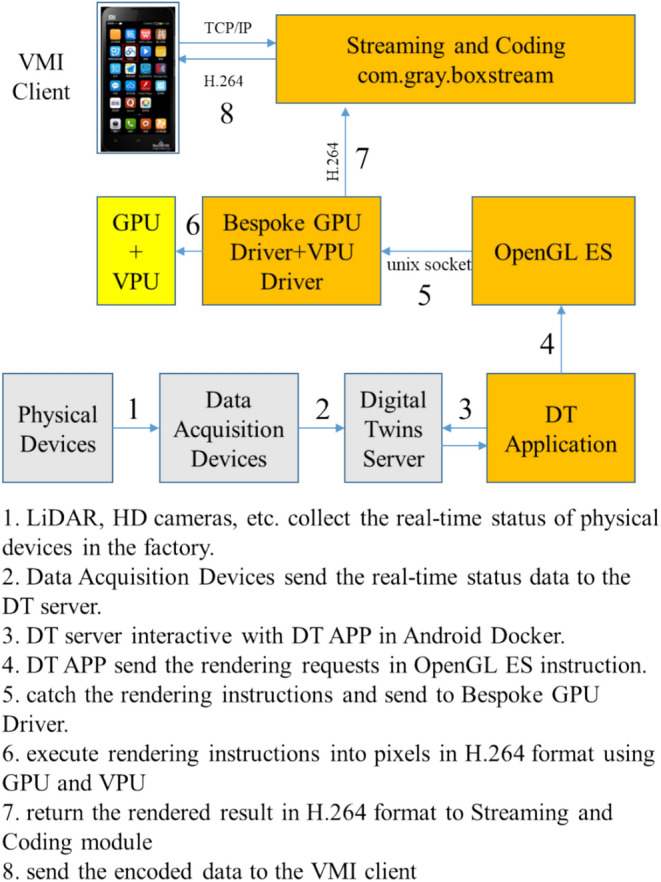
The improved VDTRIS workflow after three improvments.

After two major improving schemes were introduced, the improved workflow of the VDTRIS is shown in Fig. 6.

## Performance experiment

**Fig. 7:**
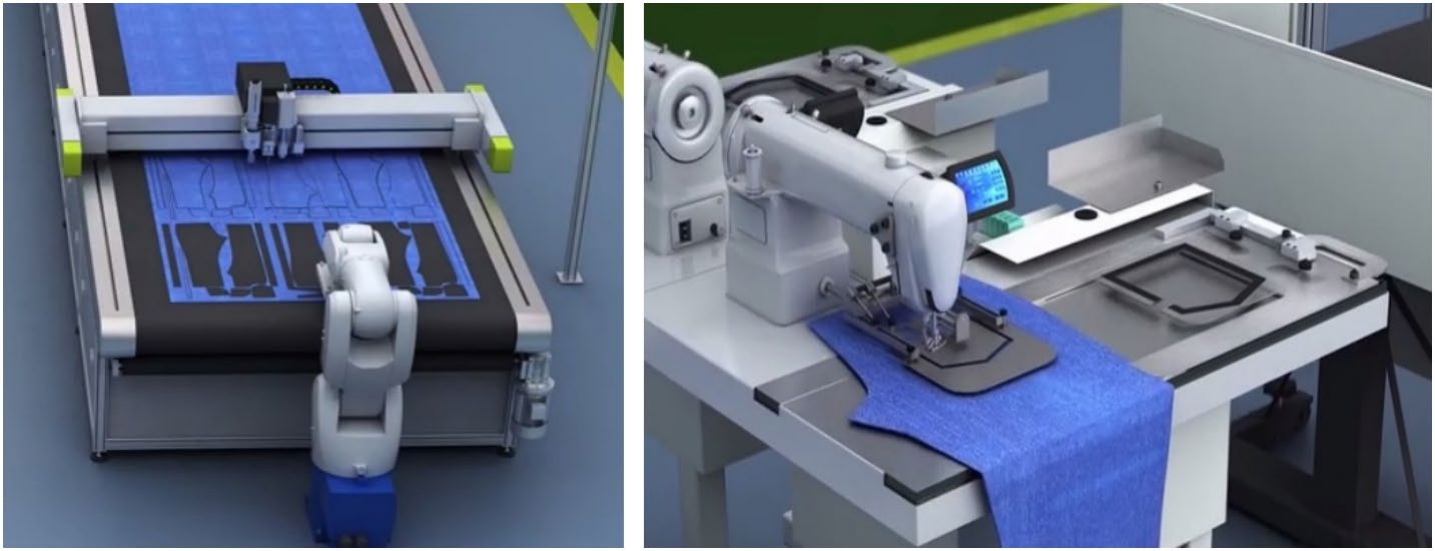
A snapshot of the garment production line digital twin system.

The Digital Twin application used in our experiments simulates a realistic garment production line monitoring scenario. The 3D scene comprises 6 distinct mechanical models, including fabric inspecting machines, robotic arms, AGVs, and sewing machines, as shown in Fig. 7. The scene features real-time animations such as conveyor belt movement, robotic arm operation, and AGV navigation. User interaction within the application includes zooming, rotating, and panning the 3D viewport, as well as clicking on equipment to query real-time status panels. This workload is representative of typical industrial DT visualization tasks, demanding sustained GPU rendering power and immediate response to interaction commands.

### Performance comparison with Robox

We compare VDTRIS with Robox (https://github.com/lag-linaro/robox), an open-source, cloud-based VMI system first released in Apr. 2018 by Huawei and Linaro. Although Robox has an architecture like VDTRIS. However, it does not support direct GPU invocation or VPU coding. In 2020, Huawei developed its commercial version named Monbox (https://www.huaweicloud.com/special/free-yunshouji-xsms.html), based on Robox. Similar to the VDTRIS, Monbox also supports direct GPU invocation, but does not have the function of VPU coding. Since Monbox is not open source, it is therefore unavailable to be accessed for evaluation publicly. So, our comparison focuses primarily on VDTRIS and Robox.

The system configuration for the comparative experiment is summarized in Table 4. A Huawei TaiShan200-2280V2 server is used for the main host server. Since the server provides 96 CPU cores and CPU utilization is calculated on a per-core basis, the theoretical maximum CPU utilization is 9600%. The VDTRIS 2.0 and Robox 2.3 are selected as the systems under comparison. A Huawei Mate70 smartphone is used as the client device.

**Table 4:** The system configuration for the comparison experiment.

	Configuration
Host server	HuaWei TaiShan200-2280v2: CPU: Kunpeng 920, 48Core *2; RAM: 512 GB; SSD: 480 GB; SATA: 4000 GB; Network: 4*GE; GPU: AMD Radeon W6800*2; VPU: Netint T432*2; Ubuntu 20.04; VDTRIS 2.0/Robox 2.3
Huawei Mate70 smartphone	HuaWei Kirin 9010 8 cores CPU, 2.3 GHz*1, 2.18 GHz*3, 1.55 GHz*4; 4 cores Maleoon 910 GPU 750 MHz; 12 GB RAM; 256 GB Flash Memory
Virtual android	CPU: 2 Core; RAM: 8 GB; Frash Memory: 64 GB; Resolution Ratio: 1920 * 1080; Frame Rate: 30 FPS; Android 11

**Fig. 8:**
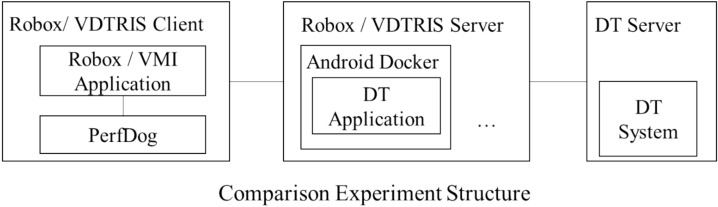
Structure of the comparative experiment.

The structure of the comparison experiment is illustrated in Fig. 8. The DT system runs on the DT server. The DT application, running inside an Android Docker on either the Robox server or the VMI server, connects to the DT server. On the client side, the Robox app or the VMI app is installed on the smartphone. When the DT application is launched on the client side, the Perfdog will record the endpoint system performance.

We focus on five key performance metrics: FPS, host-server CPU utilization, host-server RAM usage, end-to-end (E2E) latency, and DT system startup time. E2E latency is defined as the time interval from a user touch event generated on the mobile client to the corresponding visual update displayed on the screen. It includes event transmission, server-side processing, rendering, encoding, network transmission, and client-side decoding/display. DT system startup time is measured from launching the DT application to the appearance of the default UI.

**Fig. 9:**
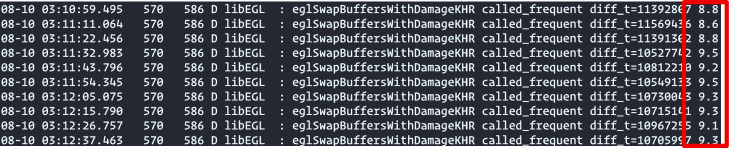
Screenshot of FPS result from the Robox-based experiment.

**Fig. 10:**
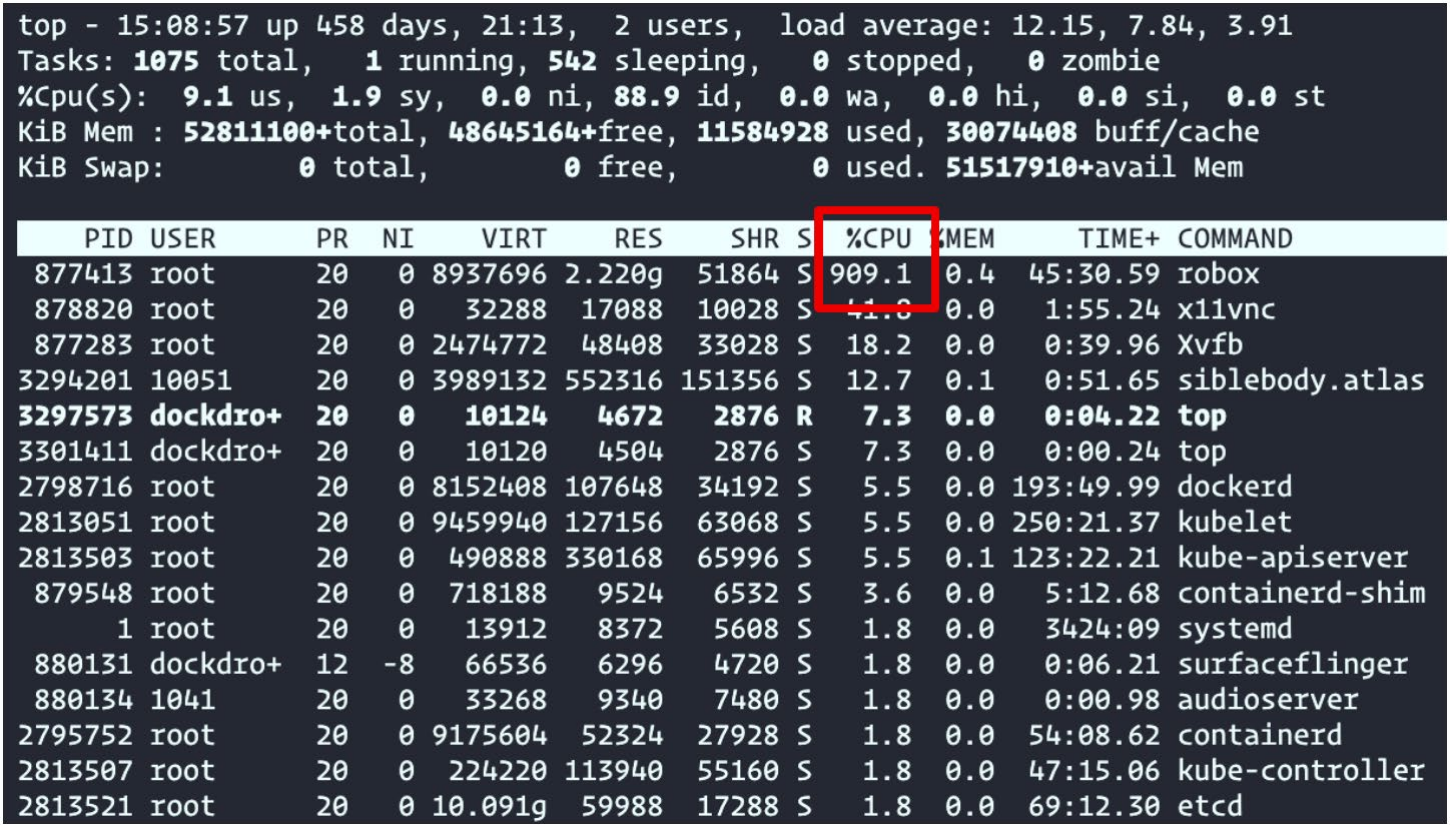
Host-server CPU utilization captured via the “top” command during the Robox-based experiment.

We repeated the experiment ten times. The mean and jitter of the monitored parameter are summarized in Table 5. As shown in Table 5, the Robox-based experiment achieves an average frame rate of 9.14 ± 0.31 FPS (Fig. 9), implying an average per-frame processing and display time of 109.4 ms. This is substantially slower than the VDTRIS-based experiment: with a mean frame rate of 31.10 FPS, VDTRIS requires only 32.2 ms per frame. Unlike VDTRIS, which can directly access GPU resources from within the Android Docker, the Robox-based system does not support direct GPU rendering or VPU coding. Instead, frames must be transferred to the host operating system for rendering, then sent back to the Android Docker, where they are encoded from RGBA to H.264 before being delivered to the client application. A large portion of CPU resources is therefore spent on data movement between the Android Docker and the host OS, which significantly increases the frame processing time. Consistently, the Robox-based experiment exhibits a much higher average host CPU utilization (928.3 ± 34.7%) than VDTRIS (20.0 ± 1.5%). Figure 10 presents a snapshot of host-server CPU utilization from the “top” command in the Robox-based experiment.

**Table 5:** The means and jitter of the monitored parameter.

Monitored parameter	Robox-based	VDTRIS-based
FPS	9.14 ± 0.31	31.10 ± 0.37
CPU utilization(%)	928.3 ± 34.7	20.0 ± 1.5
RAM usage (GB)	1.91 ± 0.13	2.03 ± 0.14
E2E latency(ms)	137.3 ± 8.9	37.7 ± 2.3
Startup time (s)	14.3 ± 1.2	27.1 ± 1.9

Because VDTRIS initializes more functional modules than Robox, it is expected to require more memory and longer initialization time. The results show that the host-server RAM usage in the Robox-based experiment is 1.91 ± 0.13 GB, about 5% smaller than that of VDTRIS (2.03 ± 0.14 GB). In terms of E2E latency, Robox-based measures 137.3 ± 8.9 ms, whereas VDTRIS-based achieves 37.7 ± 2.3 ms, reducing latency by nearly 100 ms. However, Robox-based starts up faster: its system startup time is 14.3 ± 1.2 s, approximately 1.9 × faster than VDTRIS-based. A plausible explanation is that the Robox-based system has fewer modules to load, whereas the VDTRIS-based system integrates more functions and components, leading to longer startup—an area where VDTRIS can be improved in the future.

### Performance comparison with local system

For the second experiment, we analyzed the performance of VDTRIS by comparing key parameters such as FPS, client CPU utilization, client RAM utilization, E2E latency, the required initialization time between directly running the DT client application on smartphones and running it via VMI to the Local System. The detailed description of the performance comparison experiment is as follows.

**Fig. 11:**
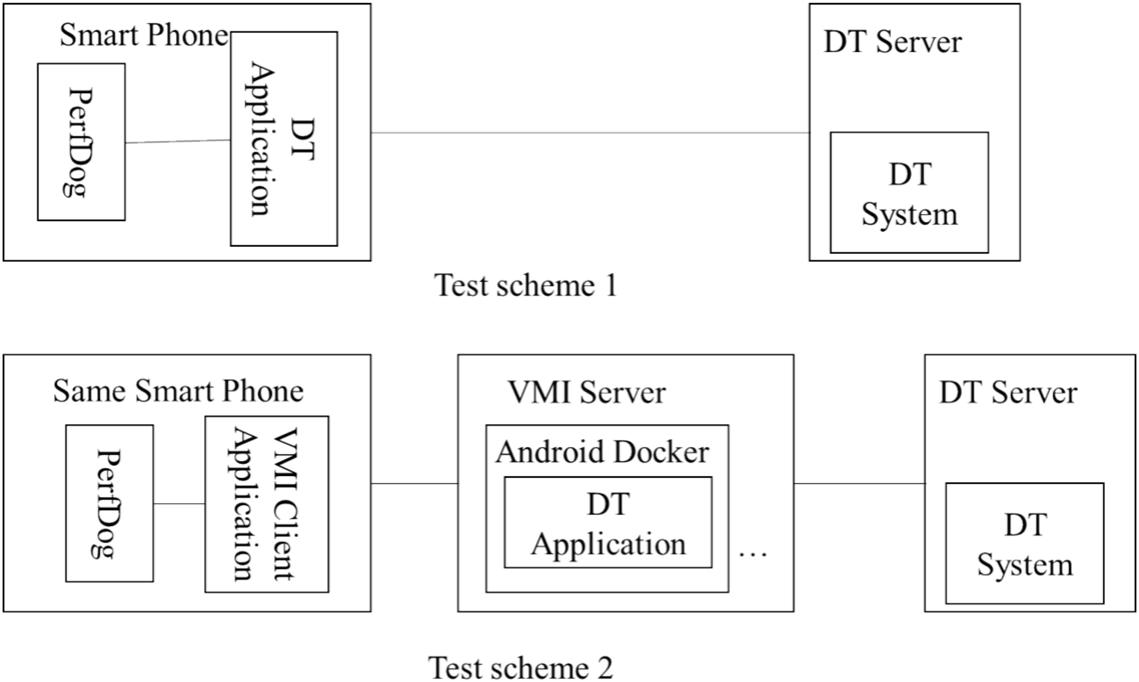
The comparison of two experimental schemes.

We deployed the DT application directly on mobile terminals and on VMI clients, corresponding to Scheme 1 and Scheme 2 as shown in Fig. 11. In Scheme 1, the Perfdog performance monitoring software is used to track performance parameters such as CPU utilization, memory usage of the mobile terminal, FPS, E2E latency, and the time required to launch the DT application. In Scheme 2, the VMI client is installed on mobile terminals with the same hardware configuration. The VMI client is connected to the VMI Server and presents the Android Docker image. Then the DT application is installed on the Android Docker. Finally, the Perfdog is used to monitor the same performance parameters as in Scheme 1.

**Fig. 12:**
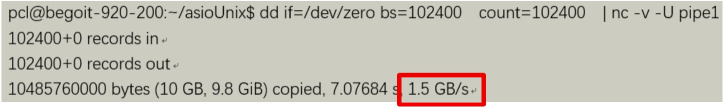
The bandwidth test result of the Unix domain socket.

**Fig. 13:**
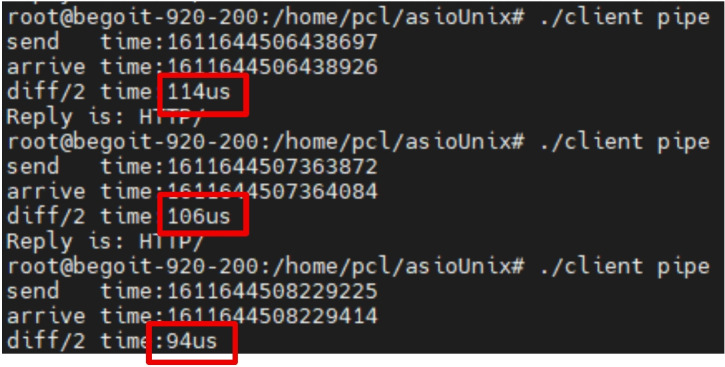
The transmission latency test result of the Unix domain socket.

The experiment is repeated ten times for each experimental scheme, and the average values of the aforementioned performance parameters are calculated. Additionally, four mobile phones with different configurations are selected for comparative testing. There is a Huawei smartphone with high-performance specifications, a Smartisan smartphone with lower-performance specifications, a Xiaomi 15 Ultra smartphone with high-performance specifications and an Honor 8X max smartphone with low-performance specifications. Their configuration parameters are detailed in Table 6. By comparing the runtime performance parameters of VDTRIS on the high-performance Huawei phone, Xiaomi phone and the lower-performance Smartisan phone, Honor phone, we can evaluate the system performance of VDTRIS when running on mobile terminals with different configuration capabilities. The configuration of Android Docker environment is as shown in Table 6. Preliminary experimental studies have shown that this configuration can smoothly run DT applications while minimizing resource consumption. Although the network interface card of the VMI server has a theoretical bandwidth of 4 GB/s, tests indicate that the Unix Domain Socket achieves an effective bandwidth of 1.5 GB/s with a transmission latency of (104.81 ± 7.26) μs, as shown in Figs. 12 and 13 below.

**Table 6:** Experimental configuration.

VMI server	HuaWei TaiShan200-2280V2: Kunpeng 920 CPU, 48Core *2; 512 GB RAM; 480 GB SSD; 4000 GB SATA; 4*GE Network; AMD Radeon W6800*2 GPU; Netint T432*2 VPU; Ubuntu 20.04; K8S 1.22.3; VDTRIS 2.0; Android 11
Huawei mate70 smart phone	HuaWei Kirin 9010 8 cores CPU, 2.3 GHz*1, 2.18 GHz*3, 1.55 GHz*4; 4 cores Maleoon 910 GPU 750 MHz; 12 GB RAM; 256 GB Flash Memory
Smartisan smart phone	Qualcomm Snapdragon 625 CPU, 8 cores, 2.0 GHz, 14 nm; Adreno 506 GPU; 4 GB RAM; 64 GB Flash Memory
Xiaomi 15 Ultra	Qualcomm Snapdragon 8 Gen 4 CPU; Adreno 830 GPU; 16 GB RAM; 512 GB Flash Memory
Honor 8X Max	Qualcomm Snapdragon 636 CPU, 8 cores; Adreno 509 GPU; 6 GB RAM; 64 GB Storage
Android docker	2 Core CPU; 8 GB RAM; 64 GB Frash Memory; 1920 * 1080 Resolution Ratio; 30 FPS Frame Rate

The experimental results are presented in Table 7. As shown in the results, on the high-performance Huawei and Xiaomi smartphone, the difference in frame rate between the two schemes is insignificant, with Scheme 2 achieving only 0.86 FPS higher than that of the local application on the Huawei phone, and 1.04 FPS higher on the Xiaomi phone.

**Table 7:** The comparison results of the experiment with Scheme 1 and Scheme 2.

		FPS	CPU utilization (%)	RAM usage (MB)	E2E latency(ms)	Startup time(s)
Huawei	Scheme 1	30.19 ± 0.41	17 ± 1.1	521 ± 17	35.8 ± 1.5	18.5 ± 1.1
	Scheme 2	31.05 ± 0.32	14 ± 0.9	673 ± 19	37.2 ± 2.1	27.8 ± 1.0
Smartisan	Scheme 1	1.60 ± 0.92	1 ± 0.26	397 ± 11	> 1000	70.1 ± 13.9
	Scheme 2	31.10 ± 0.31	16 ± 0.9	596 ± 21	38.5 ± 2.4	28.3 ± 1.1
Xiaomi	Scheme 1	30.27 ± 0.35	18 ± 0.8	580 ± 18	35.3 ± 1.7	18.9 ± 1.2
	Scheme 2	31.31 ± 0.32	13 ± 0.7	695 ± 19	37.3 ± 1.5	27.6 ± 1.3
Honor	Scheme 1	9.50 ± 0.56	83 ± 5.3	950 ± 37	112.7 ± 19.3	125.3 ± 28.7
	Scheme 2	30.8 ± 0.37	15 ± 0.8	645 ± 20	38.1 ± 2.1	28.2 ± 1.2

In terms of CPU utilization, Scheme 2 results in merely 14 ± 0.9% CPU usage on the Huawei device, 13± 0.7% CPU usage on the Xiaomi device, as it is only responsible for display and interaction functions. In contrast, Scheme 1 requires additional CPU resources to handle application launch processes alongside rendering tasks, explaining its higher CPU consumption.

For RAM usage, Scheme 2 consumes 29% more memory than Scheme 1 on the Huawei phone, 20% more memory on the Xiaomi phone, attributable to the extra memory required to run the VMI client application.

In terms of E2E latency, Scheme 2 results in merely 4% slower than Scheme 1 on the Huawei smartphone and 6% slower on the Xiaomi smartphone. This is because, on high-performance smartphones, Scheme 1 can run the DT application normally without assistance from the VMI server, and communicate directly with the DT server. As a result, Scheme 1 avoids the additional hop to the VMI server that Scheme 2 requires, leading to slightly lower latency.

Regarding startup time, Scheme 2 exhibits a 50% longer launch duration compared to the local deployment in Scheme 1 on the Huawei smartphone, and 46% longer launch duration on the Xiaomi smartphone. This can be attributed to three factors: first, the powerful hardware of the Huawei and Xiaomi devices enables rapid local application initialization; second, Scheme 2 involves cloud-based application startup with additional network transmission latency for delivering the initial image to the VMI client; and finally, the DT application can only start in the cloud after the VMI client has initialized, introducing further delay.

In summary, for high-performance terminals, implementing the VDTRIS system (Scheme 2) delivers slightly better than the non-VDTRIS approach (Scheme 1).

In contrast, due to the weaker hardware capabilities of the Smartisan smartphone, when starting the DT application locally in Scheme 1, the mean frame rate was only 1.6 FPS (625 ms per frame), the mean CPU utilization was only 1%, the mean RAM usage was 397 MB, the mean E2E latency was more than 1 s, and the mean startup time exceeded one minute. These key parameters all indicate that the DT application could not started normally in local, thereby causing abnormal phenomenon in the DT application. The poor phenomenon was also performed in Honor smartphone when starting the DT application locally in Scheme 1. The mean frame rate was 9.5 FPS (105.3 ms per frame), the mean CPU utilization was 83%, the mean RAM usage was 950MB, the mean E2E latency was 112.7ms, and the mean startup time exceeded 125.3 s. It indicates that the DT application started poorly due to the poor performance on the Honor smartphone. It can be seen that in the comparative experiment of Scheme 1, the smartphone with low configuration performs very poorly compared to the smartphone with high configuration.

In scheme 2, when starting the DT application via the VMI client on the Smartisan, the average frame rate increased rapidly by nearly 19 times, from 1.60 to 31.10. The average launch time decreased to 28.3 s, showing a substantial improvement in contrast with scheme 1. The CPU utilization increased rapidly from 1 to 16%, the RAM usage grew from 397 to 596 MB, and the E2E latency dropped rapidly from more than 1000 ms to 38.5 ms. The startup time of the VMI-based DT application was almost identical on the Huawei, Smartisan, Xiaomi, and Honor smartphones, indicating that the performance of the VDTRIS system on the client is hardly affected by the hardware configuration. VDTRIS offers a practical solution to enable devices with limited hardware resources to access applications that require substantial computational power.

Additionally, in VDTRIS, the hardware resources of Android Docker can be dynamically adjusted according to requirements, enabling VMI-based DT applications to achieve a greater adaptability. In contrast, in Scheme 1 without VDTRIS, once a smartphone is purchased, its hardware configuration is fixed. When resource requirements exceed its hardware capabilities, the smartphone cannot meet them.

## Discussion and future work

### System characteristic

The characteristics of the VDTRIS are as follows:

- Enhanced efficiency. Improvements such as Direct GPU Invocation and direct VPU Coding after GPU rendering reduce data transmission nodes and eliminate multi-stage instruction conversion. In addition, the integration of a VPU at the hardware layer to execute rendered results coding saves substantial CPU resources for other tasks.
- Superior performance. Benefiting from the powerful performance of VMI server, the VDTRIS features powerful processing capabilities, superior rendering performance, and substantial storage capabilities, enabling it to execute digital twin rendering tasks with high resource requirements. Since terminal devices are only used for display and interaction, they have low hardware resource requirements, making the system widely deployable.
- Great Adaptability. Leveraging VMI technology allows the VDTRIS application to operate seamlessly across client smartphones boasting diverse hardware setups. Moreover, Android Docker’s hardware setup offers flexible customization according to specific demands, whereas a smartphone’s hardware configuration becomes fixed once acquired, lacking the ability to adapt to changing hardware requirements.
- Easy promotion. Since the terminal devices only need low hardware resource requirements, existing Apple devices, Android devices, and H5 terminal devices can easily meet the hardware and software requirements. Therefore, the VDTRIS can be widely accepted and deployed by DT users. And this helps make full use of client users’ existing devices and reduce system investment costs.

### Limitations and future work

Experiments have shown that due to the powerful hardware configuration of Huawei and Xiaomi smartphones, the performance of DT applications installed directly on these powerful smartphones is comparable to that of the VDTRIS in respect of frame rate, CPU usage, E2E latency and RAM usage. Moreover, DT applications in local system even start faster than VDTRIS version. This indicates that if the client devices feature strong enough hardware setups, resource-demanding tasks can also be executed well. At this point, the advantages of VDTRIS are not obvious; however, there is a trade-off between hardware capabilities and financial costs, especially in underdeveloped regions.

In this paper, VMI is applied to digital twin systems. However, VMI technology has extensive adaptability across devices with varying hardware capabilities. It fully leverages the portability and ubiquity of mobile terminal devices as well as the high performance of cloud-based computing power networks. We hope that with the growing demand for machine vision and 3D image-based applications, VMI technology can be explored for application in more industries and fields.

## Conclusion

The VDTRIS proposed in this paper provides an efficient and cost-effective approach for the presentation and interaction of digital twin systems on mobile terminals. It improves the transmission and interaction performance through the following two key improvements upon a typical VMI: developing a mobile GPU driver for Android Docker to invoke GPU resources directly; and adding a VPU module to encode the rendered results into H.264 format directly. These two major improvements reduce transmission latency and CPU resource consumption. After that the VDTRIS improves the efficiency of real-time interaction and presentation of the DT application on mobile terminals.

By adopting VMI technology, VDTRIS can effectively conquer challenges including geographical restrictions, mobile devices’ insufficient computing capability, and inadequate funding. Experimental results show that VDTRIS can be deployed on terminal devices with different hardware capabilities, overcoming the problem of limited hardware capabilities of mobile terminals.

Finally, since the VDTRIS supports 3D image display and interaction across various devices, its usage can be easily expanded to other domains including Virtual Reality and Augmented Reality.

## Data Availability

The early version of the VMI is hosted open source in the following address: https://github.com/DockDroid/openvmi. The improvements presented in this paper have been integrated into the commercial version of OpenVMI and deployed in industrial products of Jiangsu Beigong Intelligent Technology Co., Ltd. It is planned to be released as open source on GitHub in the near future.
